# Utilizing a domain-specific large language model for LI-RADS v2018 categorization of free-text MRI reports: a feasibility study

**DOI:** 10.1186/s13244-024-01850-1

**Published:** 2024-11-22

**Authors:** Mario Matute-González, Anna Darnell, Marc Comas-Cufí, Javier Pazó, Alexandre Soler, Belén Saborido, Ezequiel Mauro, Juan Turnes, Alejandro Forner, María Reig, Jordi Rimola

**Affiliations:** 1 BCLC Group, Radiology Department, Hospital Clínic of Barcelona, IDIBAPS, Barcelona, Spain; 2 Computer Science, Applied Mathematics and Statistics Department, University of Girona, Girona, Spain; 3 Information Technology Department, Spanish Association for the Study of the Liver, Madrid, Spain; 4 BCLC Group, Fundació Clínic per la Recerca Biomèdica—IDIBAPS, Barcelona, Spain; 5 BCLC Group, Liver Unit, Hospital Clínic of Barcelona, Fundació Clínic per a la Recerca Biomédica (FCRB), IDIBAPS, University of Barcelona, Barcelona, Spain; 6 Centro de Investigación Biomédica en Red de Enfermedades Hepáticas y Digestivas (CIBERehd), Barcelona, Spain; 7 Gastroenterology and Hepatology, Pontevedra University Hospital Complex, Pontevedra, Spain; 8 Galicia Sur Health Research Institute, Vigo, Spain

**Keywords:** Hepatocellular carcinoma, Natural language processing, Radiology, Report, Standardization

## Abstract

**Objective:**

To develop a domain-specific large language model (LLM) for LI-RADS v2018 categorization of hepatic observations based on free-text descriptions extracted from MRI reports.

**Material and methods:**

This retrospective study included 291 small liver observations, divided into training (*n* = 141), validation (*n* = 30), and test (*n* = 120) datasets. Of these, 120 were fictitious, and 171 were extracted from 175 MRI reports from a single institution. The algorithm’s performance was compared to two independent radiologists and one hepatologist in a human replacement scenario, and considering two combined strategies (double reading with arbitration and triage). Agreement on LI-RADS category and dichotomic malignancy (LR-4, LR-5, and LR-M) were estimated using linear-weighted κ statistics and Cohen’s κ, respectively. Sensitivity and specificity for LR-5 were calculated. The consensus agreement of three other radiologists served as the ground truth.

**Results:**

The model showed moderate agreement against the ground truth for both LI-RADS categorization (κ = 0.54 [95% CI: 0.42–0.65]) and the dichotomized approach (κ = 0.58 [95% CI: 0.42–0.73]). Sensitivity and specificity for LR-5 were 0.76 (95% CI: 0.69–0.86) and 0.96 (95% CI: 0.91–1.00), respectively. When the chatbot was used as a triage tool, performance improved for LI-RADS categorization (κ = 0.86/0.87 for the two independent radiologists and κ = 0.76 for the hepatologist), dichotomized malignancy (κ = 0.94/0.91 and κ = 0.87) and LR-5 identification (1.00/0.98 and 0.85 sensitivity, 0.96/0.92 and 0.92 specificity), with no statistical significance compared to the human readers’ individual performance. Through this strategy, the workload decreased by 45%.

**Conclusion:**

LI-RADS v2018 categorization from unlabelled MRI reports is feasible using our LLM, and it enhances the efficiency of data curation.

**Critical relevance statement:**

Our proof-of-concept study provides novel insights into the potential applications of LLMs, offering a real-world example of how these tools could be integrated into a local workflow to optimize data curation for research purposes.

**Key Points:**

- Automatic LI-RADS categorization from free-text reports would be beneficial to workflow and data mining.
- LiverAI, a GPT-4-based model, supported various strategies improving data curation efficiency by up to 60%.
- LLMs can integrate into workflows, significantly reducing radiologists’ workload.

**Graphical Abstract:**

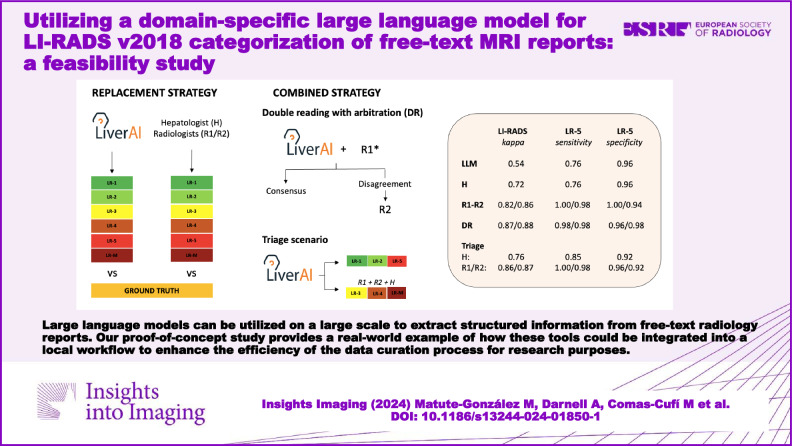

## Introduction

Structured reporting has been thought to be the key to improving clinical and radiological workflow [1, 2]. Specifically, in the field of liver cancer, the liver imaging reporting and data system (LI-RADS) was developed over a decade ago as a standardized system to assist in reporting, managing, and data collection on liver observations in patients at risk of hepatocellular carcinoma (HCC) [3]. The LI-RADS provides an algorithmic framework where liver observations are categorized based on combined major and optional ancillary features, indicating the likelihood of HCC or malignancy, and guiding subsequent imaging workup [4]. Beyond its clinical implications, LI-RADS aims to provide a standardized framework for radiology reporting to enhance the efficiency of data extraction and pooling, currently challenged by the heterogeneous terminology used in the published literature [5]. Standardized systems like LI-RADS, coupled with structured reporting, may streamline data mining from imaging reports, enabling the generation of large-scale databases essential for research and machine learning applications. Despite the growing evidence of its potential benefits, the implementation of structured reporting in clinical routine is still lacking [6], and data extraction from free-text radiology reports remains a time-consuming manual task.

Natural language processing (NLP) is a field of artificial intelligence (AI) focused on developing algorithms and models enabling machine-learning systems to interpret human language. With the generalization of large language models (LLMs) through the recent release of OpenAI’s generative pre-trained transformers (GPT), NLP has gained significant attention among researchers across various medical fields [7, 8]. In radiology, these models have demonstrated suitability for a variety of tasks, such as imaging test referrals, standardizing and simplifying radiology reports, aiding in differential diagnoses, and generating impressions [8–12]. Recent exploratory studies have also shown that these LLMs can be utilized on a large scale to extract structured and quantitative information from narrative radiology reports [13, 14]. While the potential applications of NLP in oncology had been previously explored [15], prior studies focused on customized NLP systems tailored for specific applications and trained on human-labelled datasets [16]. In contrast, new avenue LLMs are trained on unlabelled data and can be applied to complex tasks with minimal task-specific information.

This exploratory study aims to assess the performance of LiverAI, a context-based chatbot built on GPT-4 architecture, in the LI-RADS v2018 categorization of hepatic observations based on free-text radiological descriptions extracted from real-life magnetic resonance imaging (MRI) reports. To this aim, we compared the algorithm’s performance against human readers with different levels of experience. Additionally, we assessed its optimal integration into the data curation process, considering both an add-on scenario and the sequential use of the algorithm in a triage scenario.

## Material and methods

The study protocol was approved by the Clinical Research Ethics Committee from our institution (HCB/2023/0900). Informed consent was waived due to the retrospective character of data collection and the use of anonymized data.

### Data source

We randomly selected a sample of 175 free-text MRI reports from cirrhotic patients previously enroled in two post-hoc studies from a prospective series [17, 18]. The aim of these studies does not overlap with the current study. All participants had liver cirrhosis and underwent liver MRI after the detection of a new small focal liver lesion (< 2 cm) during screening ultrasound (US) for HCC. The MRI was performed using a standard protocol with extracellular gadolinium, including all four phases, aiming to characterize the lesions identified. Original reports, authored between 2004 and 2015, were written in Spanish by 15 different radiologists from our institution and did not include any mention of LI-RADS categorization. All reports were reviewed and codified by the study coordinator (M.M.-G.) for inclusion in the study database. The free-text descriptions of the lesions of interest were manually extracted, and the radiologist’s final impressions were eliminated to prevent biases in the algorithm or human readers (i.e. consistent with HCC, etc.). A total of 13 reports were excluded due to missing measurements, the absence of LI-RADS features, or the reporting of artefacts hindering proper evaluation. The dataset included 162 reports with a total of 171 observations, comprising a wide variety of reporting styles and lexicon.

### Development of the domain-specific chatbot

**Fig. 1:**
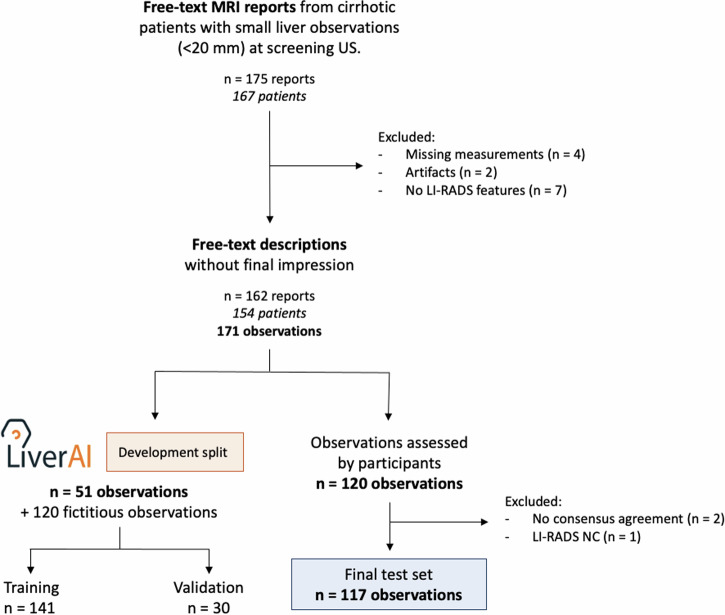
Flowchart of study design

The extracted observations were randomly divided into a training set (*n* = 51) and a test set (*n* = 120). Given the limited number of genuine descriptions, our priority was to maximize the size of the test set to rigorously evaluate the model’s performance. To enhance the size and diversity of the training dataset, we developed a specialized script using OpenAI’s GPT-4 API model to generate an additional 120 fictitious examples. These synthetic cases were carefully reviewed and corrected by the study coordinator before their inclusion in the development cohort, ensuring their accuracy and relevance. In total, the final training set comprised 141 liver observations (51 real and 90 synthetic), with an additional 30 descriptions designated for validation (Fig. 1). The study coordinator annotated the training dataset.

The LLM evaluated in this study was LiverAI, a domain-specific chatbot sponsored by the Spanish Society of Liver Diseases (AEEH from its acronym in Spanish). LiverAI was developed on GPT-4 architecture between November 2023 and January 2024 using OpenAI’s application programming interface (API). The development process of LiverAI involved a series of iterative experiments and optimizations to enhance its performance (Appendix S1). Initially, we manually transformed the primarily graphical and tabular LI-RADS data into AI-optimized textual descriptions [19]. Text information was sourced from the publicly available American College of Radiology (ACR) CT/MRI LI-RADS v2018 Core (https://www.acr.org/-/media/ACR/Files/RADS/LI-RADS/LI-RADS-2018-Core.pdf). The model was fine-tuned and embeddings were applied to enhance its categorization capabilities within the LI-RADS system, complemented by prompt engineering to further refine its performance. Scripts, along with all relevant documentation, have been made available on GitHub under the Apache 2.0 License (https://github.com/aeehliver/lirads). Finally, to assess and validate this approach, we compared the performance of LiverAI against that of publicly available generic chatbots GPT-3.5 and GPT-4 (Appendix S3 and Table S1).

### Study procedures and ground truth definition

**Fig. 2:**
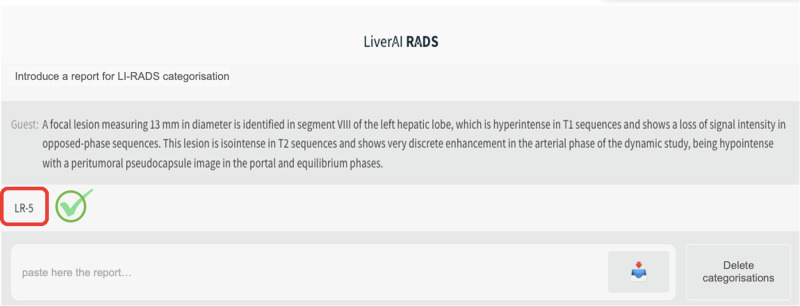
Chatbot interface of the domain-specific LLM (LiverAI), showing an example of a free-text liver observation description correctly categorized. Both the chatbot interface and the report have been translated from Spanish to English for understandability reasons. LI-RADS, liver imaging reporting and data system

Five independent radiologists, including the study coordinator, familiar with the LI-RADS system and with varying levels of experience in liver imaging were invited to participate in the study: M.M.-G. (2 years), A.D. (25 years), A.S. (5 years), B.S. (3 years), and J.R. (20 years). Additionally, an expert hepatologist with 9 years of experience (E.M.) was also included. Each reader independently assigned a LI-RADS category to all liver observations included in the test set (*n* = 120), based on the narrative report descriptions and according to the current LI-RADS v2018 definition. The same report information was presented in a one-shot query to the chatbot by the study coordinator, who registered the output given by the algorithm (Fig. 2).

**Fig. 3:**
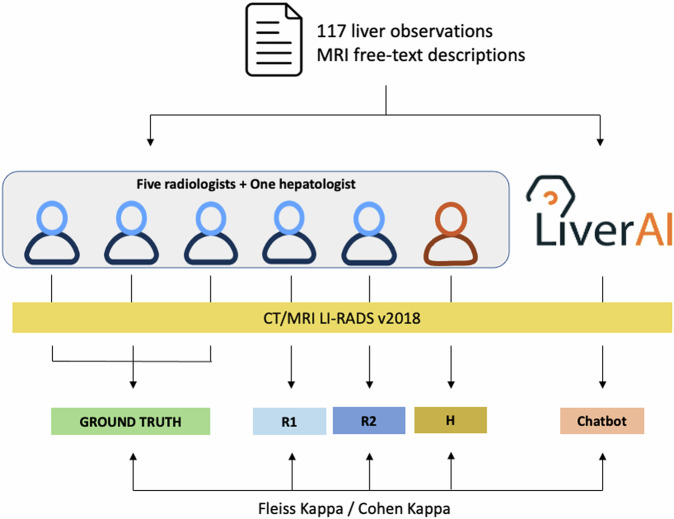
Workflow and performance analysis of the domain-specific LLM (LiverAI). LI-RADS, liver imaging reporting and data system; R1 and R2, independent radiologist 1 and 2, respectively; H, hepatologist

The ground truth for each observation was established through a majority assessment consensus among three of the radiologists (M.M.-G., A.D., and A.S.). The performances of the two remaining independent radiologists (B.S. and J.R.) and the hepatologist (E.M.), who were not part of the ground truth definition, were evaluated and compared with LiverAI, using the ground truth as the reference standard (Fig. 3). Examples of correct and incorrect categorizations made by the chatbot are provided in the online supplement (Table S2).

### Clinical scenarios

**Fig. 4:**
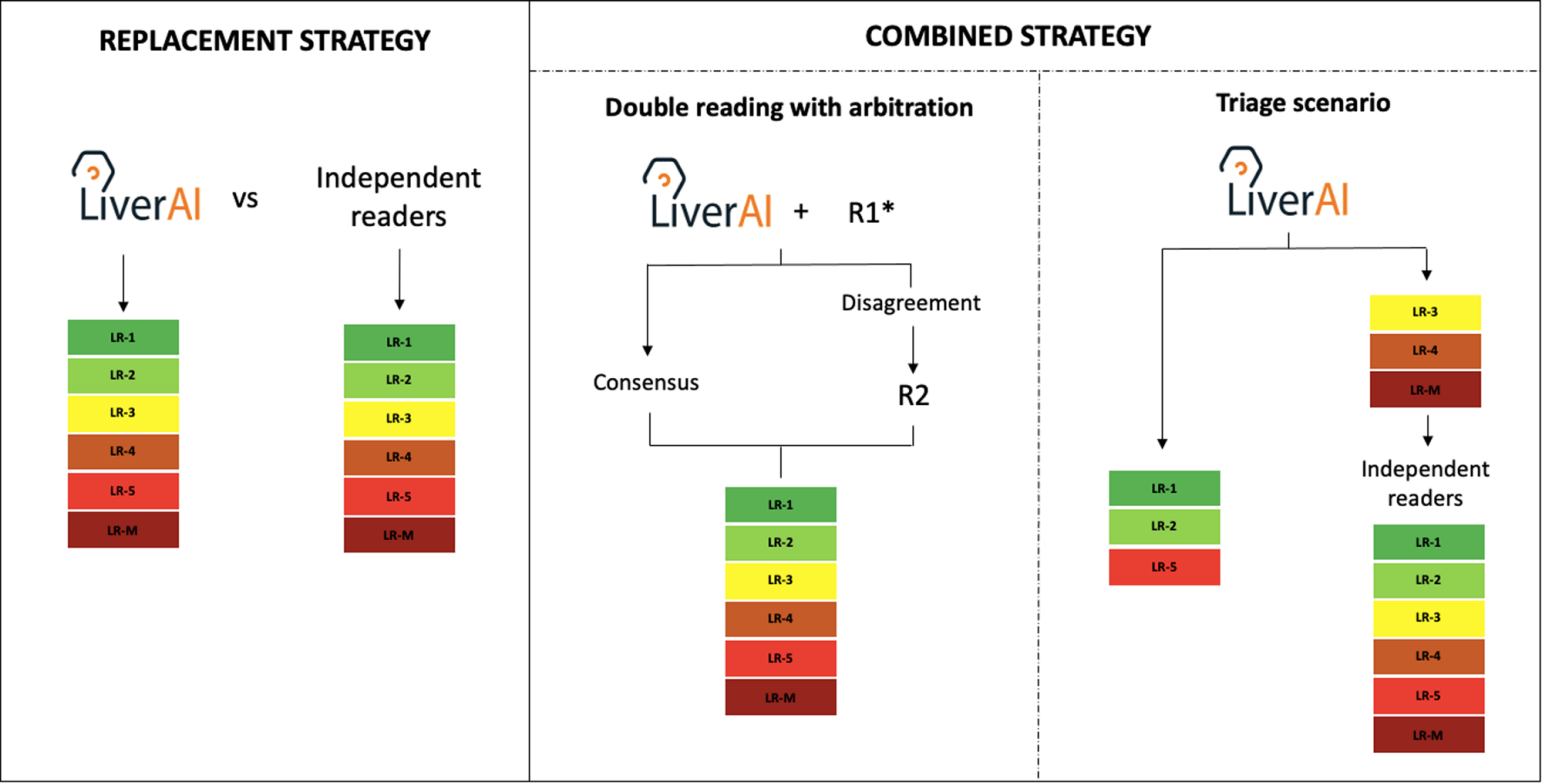
The clinical scenarios evaluated to assess the optimal integration of the domain-specific LLM (LiverAI) for LI-RADS categorization of liver observations described in free-text MRI reports. R1 and R2, independent radiologists 1 and 2, respectively. * The double reading strategy was repeated considering both the combination of R1 and LiverAI, with posterior arbitration by R2; and the combination of R2 and LiverAI, with arbitration by R1

To explore the optimal integration of the developed algorithm into the data curation process, three different scenarios were investigated (Fig. 4). On the one hand, we considered the algorithm as a potential replacement for radiologists in data categorization (replacement strategy). On the other hand, we explore the sequential performances of the LLM algorithm and the independent readers using two combined strategies. In the first, we evaluated the performance of the combination of either Radiologist 1 (R1, B.S.) or Radiologist 2 (R2, J.R.) with the chatbot, with the other participant serving as an arbitrator to resolve discrepancies (R1 + LiverAI#R2, or R2 + LiverAI#R1) (double reading with arbitration strategy). In the second, the chatbot assessed all liver observations, and the readers, including the hepatologist (H, E.M.), proceeded to evaluate only those observations categorized as LR-3, LR-4, and LR-M by the algorithm (triage strategy). The primary endpoint of the three explored approaches was the accuracy of LI-RADS categorization. The secondary endpoint was the proportion of LR-5 observations detected.

### Statistical analysis

Agreement across LI-RADS categories was computed using two different approaches. First, considering each LI-RADS category separately, ordered by ascending risk of malignancy [20]: LR-1, LR-2, LR-3, LR-4, LR-5, and LR-M. Second, using a binary approach in which categories were dichotomized as lower-risk of malignancy or probably/definitely malignant (LR-1, LR-2, and LR-3 vs LR-4, LR-5, and LR-M).

The three independent readers and chatbot’s performance in the final test set, along with the resulting categorizations from each combined strategy, were evaluated according to the consensus ground truth. Types of disagreements were depicted in confusion matrices. Agreement against the ground truth across all LI-RADS categories was assessed using weighted kappa with linear weighting, whereas the dichotomized approach was measured through Cohen’s kappa. Fleiss’ kappa was computed for agreement among more than two readers. Based on kappa coefficients, agreement was categorized as follows: < 0.20, slight; 0.21–0.40, fair; 0.41–0.60, moderate; 0.61–0.80, substantial; and > 0.80, almost perfect [21]. The sensitivity and specificity for LR-5 were also calculated. Confidence intervals (CI) were obtained using bootstrap resampling, except for the kappa indices where the binomial normal approximation was used. All statistical analyses were computed using the *R* statistical software (version 4.2.2).

## Results

### Patient and observation characteristics

Figure 1 shows the flowchart of reports included in the study. A total of 291 liver observations were included: 120 were fictitious, and 171 were extracted from liver MRI reports of 154 patients. Table 1 summarizes the characteristics of each dataset.

**Table 1:** Characteristics of the liver observations included in the test and development datasets

Characteristic	Training set, (*n* = 141)	Validation set, (*n* = 30)	Test set, (*n* = 120)
Age at diagnosis (years), mean (range)*	61 (41–81)	–	65 (43–84)
Sex*			
Female	14 (29.8)	–	47 (41.7)
Male	33 (70.2)		60 (58.3)
LI-RADS major features**			
Size (mm), median (IQR)	15 (11–18)	14.5 (11–17)	15 (12–18)
Nonrim APHE	79 (56.0)	13 (43.3)	99 (82.5)
Nonperipheral “washout”	53 (37.6)	9 (30.0)	70 (58.3)
Enhancing “capsule”	31 (22.0)	4 (13.3)	39 (32.5)
LI-RADS version 2018 categories			
LR-1	19 (13.5)	5 (16.7)	1 (0.8)
LR-2	20 (14.2)	5 (16.7)	7 (5.8)
LR-3	30 (21.3)	5 (16.7)	33 (27.5)
LR-4	22 (15.6)	5 (16.7)	5 (4.2)
LR-5	34 (24.1)	5 (16.7)	66 (55.0)
LR-M	16 (11.3)	5 (16.7)	5 (4.2)
LR-NC	0 (0)	0 (0)	3 (2.5)

*APHE* arterial phase hyperenhancement, *IQR* interquartile range, *LR-NC* LI-RADS not categorisable

* Data for liver observations extracted from genuine MRI reports. There were 171 observations in 154 patients

** Threshold growth is not considered, as the MRI examinations included were performed to characterize liver observations identified through screening US, without follow-up assessments

The initial test dataset comprised MRI descriptions of 120 liver observations. Based on the ground truth, 1 observation was classified as LR-1 (0.8%), 7 as LR-2 (5.8%), 33 as LR-3 (27.5%), 5 as LR-4 (4.2%), 66 as LR-5 (55%), and 5 as LR-M (4.2%). There was no report classified as observation with tumor-in-vein present (LR-TIV). One observation was classified as “not categorisable” (LR-NC), and there was no consensus agreement for two observations. These three observations were excluded from the performance analysis in the final test dataset (*n* = 117) (Fig. 1).

### Agreement among human readers

**Fig. 5:**
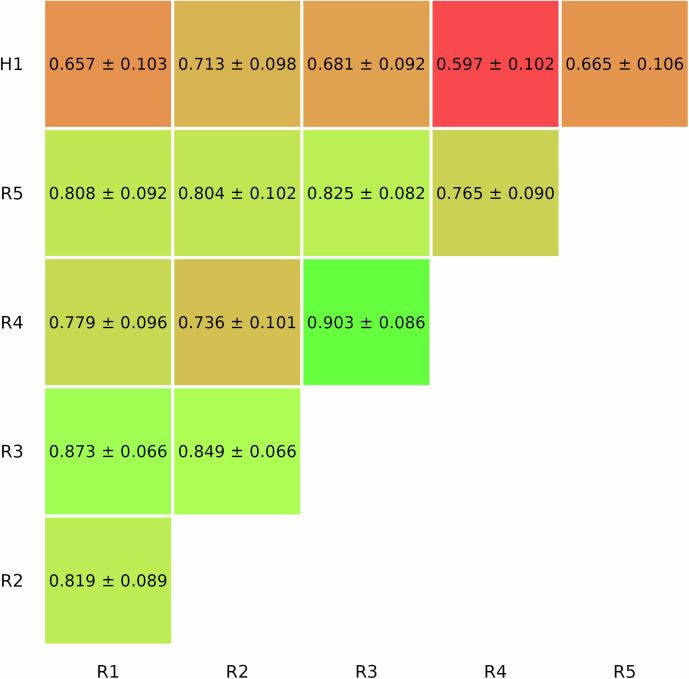
Inter-reader agreement across LI-RADS categories among all human readers. R, readers

The five radiologists agreed on the LI-RADS categorization of 70.0% (84 of 120) liver observations, with substantial inter-reader agreement (κ = 0.74 [95% CI: 0.68–0.79]). For dichotomized malignancy, agreement was almost perfect (κ = 0.87 [95% CI: 0.79–0.92]), with a percentage agreement of 105/120 (87.5%). The hepatologist’s agreement with each radiologist was consistently lower than the agreement among radiologists when considering all possible combinations. The inter-reader agreement values between pairs across all LI-RADS categories are summarized in Fig. 5.

### Optimal integration of LiverAI on data mining strategy

#### Performance of LiverAI compared with independent readers (replacement strategy)

**Fig. 6:**
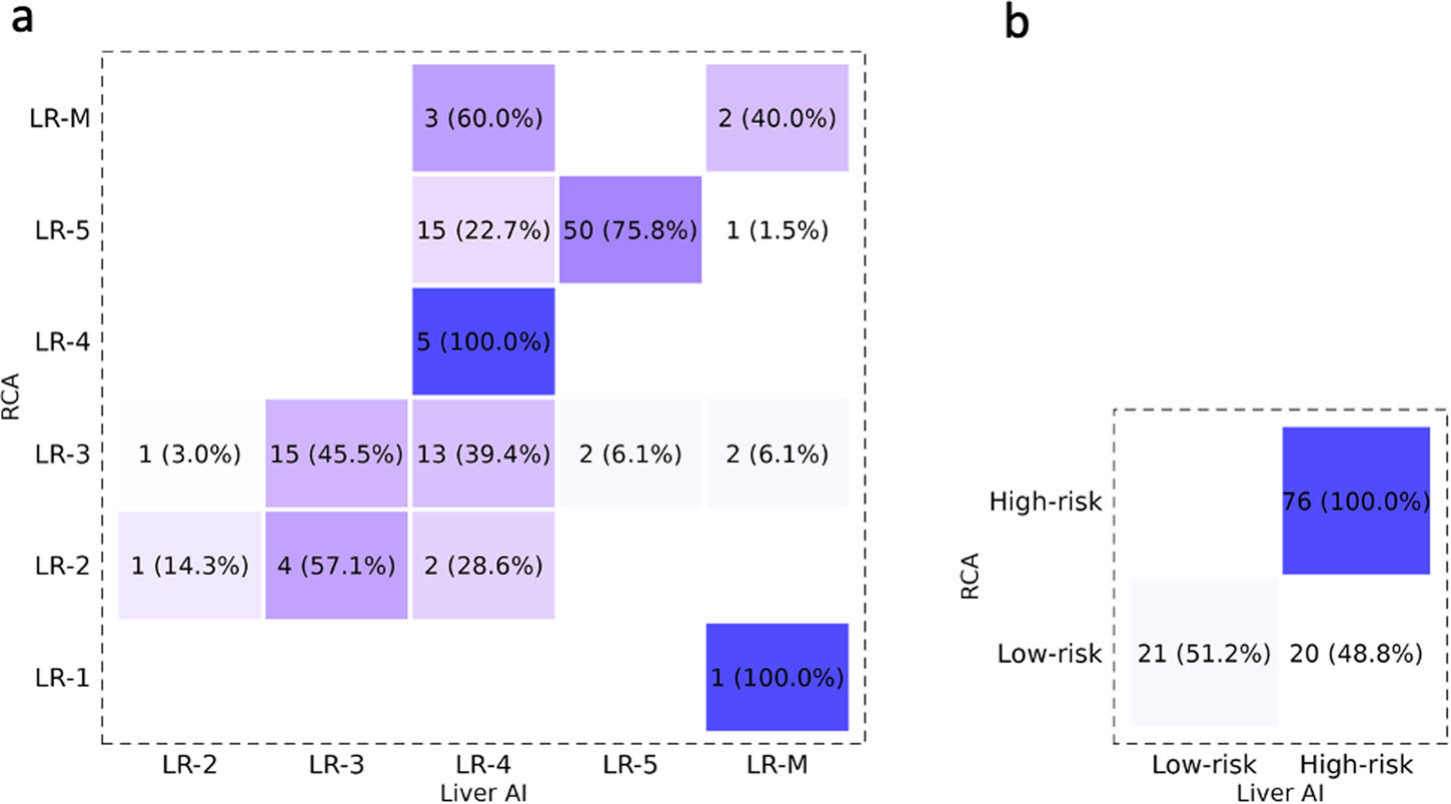
Assessment by LiverAI is compared to the consensus radiologic assessment across all LI-RADS categories (**a**) and dichotomized malignancy (**b**). RCA, radiologic consensus assessment

According to the ground truth, 73/117 (62.4%) observations were accurately categorized by the algorithm (Fig. 6a), with moderate agreement against the established consensus (κ = 0.54 [95% CI: 0.42–0.65]). Sensitivity and specificity for LR-5 were, respectively, 0.76 (95% CI: 0.69–0.86) and 0.96 (95% CI: 0.91–1.00). Regarding independent readers, 101/117 (86.3%) and 105/117 (89.7%) observations were correctly categorized by Radiologist 1 (R1) and Radiologist 2 (R2). Agreement against the consensus was almost perfect for all LI-RADS categories (R1: κ = 0.82 [95% CI: 0.73–0.91]; and R2: κ = 0.86 [95% CI: 0.77–0.95]), significantly higher than that of the model. Sensitivity and specificity for LR-5 were, respectively, 1.00 (95% CI: 1.00–1.00) and 1.00 (95% CI: 1.00–1.00) for R1, and 0.98 (95% CI: 0.95–1.00) and 0.94 (95% CI: 0.87–1.00) for R2. By contrast, 85/117 (72.6%) observations were correctly categorized by the hepatologist. Agreement with the ground truth was substantial (κ = 0.72, 95% CI: 0.62–0.81), but not significantly better compared to the model, and significantly lower in accuracy than that of the most experienced radiologist (R2). Sensitivity and specificity for LR-5 were, respectively, 0.76 (95% CI: 0.66–0.86) and 0.96 (95% CI: 0.90–1.00).

For dichotomized malignancy, the algorithm correctly categorized 97/117 (82.9%) observations (Fig. 6b), with moderate agreement against the ground truth (κ = 0.58 [95% CI: 0.42–0.73]). Sensitivity and specificity for probably/definitely malignant observations (LR-4, LR-5, and LR-M) were, respectively, 1.00 (95% CI: 1.00–1.00) and 0.51 (95% CI: 0.37–0.66). The agreement of the three independent readers was significantly higher (detailed statistics in Table 2).

**Table 2:** Performance statistics in the test dataset for independent human readers and the domain-specific LLM (LiverAI) standalone, and in the two combined scenarios (double reading with arbitration for radiologists, and triage for all participants)

Strategy	LI-RADS		LR-5			Dichotomized LI-RADS		Malignancy	
	Accuracy	*K* value	Accuracy	Sensitivity	Specificity	Accuracy	*K* value	Sensitivity	Specificity
Radiologist 1	0.86 (0.81–0.92)	0.82 (0.73–0.91)	1.00 (1.00–1.00)	1.00 (1.00–1.00)	1.00 (1.00–1.00)	0.97 (0.92–0.99)	0.92 (0.85–1.00)	1.00 (1.00–1.00)	0.90 (0.82–0.97)
Radiologist 2	0.90 (0.86–0.96)	0.86 (0.77–0.95)	0.97 (0.93–0.99)	0.98 (0.95–1.00)	0.94 (0.87–1.00)	0.96 (0.90–0.98)	0.91 (0.82–0.99)	0.99 (0.96–1.00)	0.90 (0.81–0.99)
Hepatologist	0.73 (0.66–0.83)	0.72 (0.62–0.81)	0.85 (0.80–0.91)	0.76 (0.66–0.86)	0.96 (0.90–1.00)	0.94 (0.90–0.97)	0.87 (0.77–0.96)	0.96 (0.92–1.00)	0.90 (0.81–0.98)
LiverAI	0.62 (0.55–0.71)	0.54 (0.42–0.65)	0.85 (0.80–0.91)	0.76 (0.69–0.86)	0.96 (0.91–1.00)	0.83 (0.75–0.89)	0.58 (0.42–0.73)	1.00 (1.00–1.00)	0.51 (0.37–0.66)
Pairs #1	0.92 (0.88–0.97)	0.87 (0.79–0.96)	0.97 (0.94–1.00)	0.98 (0.95–1.00)	0.96 (0.91–1.00)	0.97 (0.94–1.00)	0.92 (0.85–1.00)	1.00 (1.00–1.00)	0.90 (0.81–0.99)
Pairs #2	0.91 (0.87–0.96)	0.88 (0.80–0.96)	0.98 (0.96–1.00)	0.98 (0.95–1.00)	0.98 (0.94–1.00)	0.97 (0.95–1.00)	0.94 (0.88–1.00)	1.00 (1.00–1.00)	0.93 (0.83–1.00)
Triage #R1	0.87 (0.82–0.92)	0.86 (0.79–0.93)	0.98 (0.96–1.00)	1.00 (1.00–1.00)	0.96 (0.91–1.00)	0.97 (0.95–1.00)	0.94 (0.88–1.00)	1.00 (1.00–1.00)	0.93 (0.84–1.00)
Triage #R2	0.89 (0.84–0.95)	0.87 (0.79–0.94)	0.96 (0.92–0.99)	0.98 (0.95–1.00)	0.92 (0.84–0.99)	0.96 (0.93–0.99)	0.91 (0.82–0.99)	0.99 (0.96–1.00)	0.90 (0.81–0.99)
Triage #H	0.77 (0.71–0.85)	0.76 (0.67–0.84)	0.88 (0.82–0.93)	0.85 (0.78–0.92)	0.92 (0.84–0.98)	0.94 (0.90–0.97)	0.87 (0.77–0.96)	0.97 (0.94–1.00)	0.88 (0.78– 0.95)

Data in parentheses are 95% CIs. Pairs #1, double reading by reader 1 and LiverAI, with posterior arbitration by reader 2; Pairs #2, double reading by reader 2 and LiverAI, with posterior arbitration by reader 1; Triage #1, Triage #2 and Triage #H indicate triage by LiverAI with posterior assessment by radiologist 1, radiologist 2, and the hepatologist, respectively

#### Double reading with arbitration strategy

The combination of either R1 or R2 with the chatbot, with posterior arbitration by the other human participant (R1 + LiverAI#R2, or R2 + LiverAI#R1), resulted in an almost perfect agreement for both strategies, with no statistical significance when compared with each human reader’s individual performance (R1 + LiverAI#R2: κ = 0.87 [95% CI: 0.79–0.96]; and R2 + LiverAI#R1: κ = 0.88 [95% CI: 0.80–0.96]). Accordingly, 107/117 (91.5%) and 106/117 (90.6%) observations were accurately categorized, with a sensitivity and specificity for LR-5 of 0.98 (95% CI: 0.95–1.00) and 0.96 (95% CI: 0.91–1.00) for the first combination, and 0.98 (95% CI: 0.95–1.00) and 0.98 (95% CI: 0.94–1.00) for the second. Similarly, no difference in the agreement was observed for dichotomized malignancy (Table 2). By employing this approach, one of the readers assessed the whole dataset, while the arbitrators decreased their workload from 120 observations to 57 (− 52.5%) (R2) and 48 (− 60.0%) (R1).

#### Triage strategy

The sequential assessment by R1 and R2 of the observations categorized as LR-3, LR-4 and LR-M by the algorithm led to an almost perfect agreement for both radiologists (R1: κ = 0.86 [95% CI: 0.79–0.93]; and R2: κ = 0.87 [95% CI: 0.79–0.94]), with no statistically significant difference when compared with each reader’s individual performance. Following this strategy, 102/117 (87.2%) and 104/117 (88.9%) observations were accurately categorized for both readers, with a sensitivity and specificity for LR-5 of 1.00 (95% CI: 1.00–1.00) and 0.96 (95% CI: 0.91–1.00) for R1, and 0.98 (95% CI: 0.95–1.00) and 0.92 (95% CI: 0.84–0.99) for R2 (Table 2). Regarding the hepatologist, 90/117 (76.9%) observations were accurately categorized with this strategy. Although there were no statistically significant differences compared to the individual performance (Table 2), this approach slightly improved agreement with the ground truth (κ = 0.76 [95% CI: 0.67–0.84]), and sensitivity (0.85 [95% CI: 0.78–0.92]).

For dichotomized malignancy, no difference in the agreement was observed, showing all readers almost perfect agreement against the ground truth (Table 2). With this strategy, the workload for each reader decreased from 120 observations to 66 (− 45%).

## Discussion

In this proof-of-concept study, we assess the feasibility of employing a locally run domain-specific LLM, LiverAI, for LI-RADS category annotation of unlabelled free-text MRI reports of cirrhotic patients with newly detected liver observations. Our results suggest that our model can be used efficiently for this purpose, potentially reducing the workload of expert radiologists when integrated into the human workflow.

Several recent studies have explored the ability of general-purpose LLMs to extract valuable information from reports [13, 14, 22, 23], even specifically for HCC imaging [16, 24]. In a recent study, Gu et al leveraged GPT-4 for LI-RADS feature extraction and categorization of multilingual free-text reports [25]. However, their cohort predominantly included larger liver observations, with 75% measuring over 21 mm (median size, 30 mm [interquartile range (IQR), 21–50 mm]). This introduces an inherent bias towards higher LI-RADS categories, as liver observations greater than 20 mm are already highly suspicious of HCC (almost all would be virtually categorized as at least LR-4). In contrast, our study focuses specifically on small liver observations (< 20 mm), which presents a greater challenge for LI-RADS categorization due to the increased granularity required within this size range.

Another significant strength of our study is that the analysis was not limited to a single strategy. Although the individual performance of the human radiologists was significantly better than that of LiverAI alone across all LI-RADS categories (κ = 0.82–0.86 vs 0.54), the performance of the algorithm yielded positive results regarding specificity for LR-5 identification (0.96) and sensitivity for malignant observations (1.00), which allowed a second approach considering its potential adoption as a triage tool. Thus, when the chatbot was integrated into the human workflow, both double reading with arbitration and triage strategy showed improved efficacy for overall LI-RADS categorization (κ = 0.87–0.88 vs 0.86–0.87, respectively) and dichotomized malignancy (κ = 0.92 vs 0.91–0.94, respectively), with no statistical significance when compared with the individual performance of the human readers. The high agreement with the ground truth observed in the triage strategy, coupled with the reduction in workload, makes this approach the most efficient for integrating our LLM into the data curation workflow. As the implementation of machine-learning applications to clinical practice is becoming a matter of concern for the radiological community [26], our work provides a real-world example of how local AI solutions could be integrated into clinical routine and constitute a relief for radiologists’ workload [27].

Beyond inherent limitations in model development, such as data size and quality, LiverAI’s performance may have been hindered by other factors. Experienced radiologists possess a depth of contextual understanding that allows them to interpret subtle nuances in free-text reports, which go beyond the tabular information considered by the LI-RADS. The lack of standardized LI-RADS terminology in the original reports represents an additional limitation. Since the primary recipients of this information, such as patients, clinicians, or even NLP tools, typically lack this level of expertise, the validity of free-text radiological descriptions in the information flow may be questioned. In fact, the hepatologist’s performance, despite being an expert in liver oncology, did not reach the level of the radiologists. Structured reporting and standardized systems like LI-RADS provide a homogeneous framework for radiology reporting, which not only improves communication with referring physicians [28], but also may ease automated report assessment and content extraction in big data analyses [29]. Additionally, in line with other relevant studies [30], our model was trained and tested using a single-center patient dataset with reports in the local language. According to a recent cross-language validation study, the agreement for BI-RADS category assignments between humans and LLMs varied across languages, with better results in English compared to reports written in Italian or Dutch [31]. This raises concerns about a “privilege bias”, as some geographic groups—minority languages and underdeveloped societies—may be excluded from receiving the same potential benefits of this type of AI solution [32]. A more standardized approach to structured reporting and the generalization of standardized terminologies could help mitigate this bias and enhance the generalizability of NLP models across different languages and healthcare settings.

Working with LLMs presents a major challenge in terms of patient data protection. The adoption of ChatGPT and other general-purpose LLMs in clinical practice involves transmitting health information to company servers, inevitably giving rise to privacy concerns [14, 33, 34]. LiverAI is constructed on a GPT architecture, and despite complying with local privacy-preserving regulations, all input clinical data is transmitted to OpenAI’s external server, which constitutes a limiting factor when considering its potential generalization into clinical routine.

There are other various limitations to our study. First, the limited number of radiology reports in both the training and test datasets may have reduced the potential performance of our LLM. Second, the chatbot’s output is based solely on the cross-sectional information written in the reports, which may be biased by the individual interpretation of the author radiologist, without considering additional clinical data such as pathology results or follow-up imaging. Third, as LI-RADS may evolve over time, ensuring the adaptability of these models to future updates is crucial for maintaining its relevance in clinical practice.

In conclusion, our proof-of-concept study provides novel insights into the potential applications of LLMs, offering an original approach that serves as a real-world example of how these tools could be integrated into a local workflow to increase efficiency in a research setting.

## Supplementary information

ELECTRONIC SUPPLEMENTARY MATERIAL

## Data Availability

The data that support the findings of this study are available from the corresponding author, upon reasonable request.

## Abbreviations

AI: Artificial intelligence
GPT: Generative pre-trained transformers
HCC: Hepatocellular carcinoma
IQR: Interquartile range
LI-RADS: Liver imaging reporting and data system
LLM: Large language model
MRI: Magnetic resonance imaging
NLP: Natural language processing
US: Ultrasound
