# Supplementary material for: Utilizing a domain-specific large language model for LI-RADS v2018 categorization of free-text MRI reports: a feasibility study
Source: Insights Imaging. 2024 Nov 22;15:280. doi: 10.1186/s13244-024-01850-1 (PMC11584817; doi:10.1186/s13244-024-01850-1)
Supplement: Supplementary file 1 — ELECTRONIC SUPPLEMENTARY MATERIAL [file 13244_2024_1850_MOESM1_ESM.pdf]

Utilizing a domain-specific large language model for LI-RADS v2018 categorisation of free-text MRI reports: a feasibility study

ELECTRONIC SUPPLEMENTARY MATERIAL

Appendix S1.

Experiments performed to optimize the model's performance

Our optimization process followed a scientific trial-and-error approach, exploring multiple techniques to improve the model's performance. We began by incorporating all relevant information into embeddings, which initially proved suboptimal. We then refined our approach by selectively embedding only key aspects of the LI-RADS criteria, which provided a slight improvement. Next, we attempted fine-tuning the model. The initial fine-tuning resulted in overfitting due to the limited dataset. To address this, we generated 90 additional synthetic cases, which were carefully reviewed and corrected by a human expert. These synthetic cases were then used in a second round of fine-tuning. Although this approach helped to mitigate the overfitting issue and yielded some improvements, the enhancements were still modest overall.

A crucial part of our development process involved advanced prompt engineering for the final version of LiverAI. We implemented an innovative approach where human experts first identified errors in the AI's output. Based on these human-identified errors, we developed an automated system that generated and tested variations of the prompt. This system iteratively refined the prompt, allowing the AI to create more general, rather than specific, rules to address its shortcomings without constant human intervention. The automated process continued until the

optimal prompt was achieved, significantly enhancing LiverAI's performance in accurately categorising liver observations according to LI-RADS criteria.

Importantly, we found that the most significant improvements came from the synergistic application of these various approaches. The combination of selective embeddings, careful fine-tuning, and our advanced automated prompt engineering techniques collectively contributed to the model's enhanced performance. This multi-faceted strategy allowed us to leverage the strengths of each approach while mitigating their individual limitations.

Throughout the development process, we continuously evaluated the model's performance against our test set, refining our approach based on these results. The final version of LiverAI, benefiting from this comprehensive optimization strategy, demonstrated superior performance compared to earlier iterations and generic models. Scripts, along with all relevant documentation, has been made available on GitHub under the Apache 2.0 License (https://github.com/aeehliver/lirads).

Appendix S2.

Performance of the algorithm in the validation set

In the validation dataset, 22 out of 30 (73.3%) observations were accurately categorised by the chatbot: 3 out of 5 (60%) for LR-1, 3 out of 5 (60%) for LR-2, 3 out of 5 (60%) for LR-3, 4 out of 5 (80%) for LR-4, 4 out of 5 (80%) for LR-5, and 5 out of 5 (100%) for LR-M. Among the cases where the algorithm failed, categorisations were just one category from the correct LI-RADS, except for the two failed LR-1 cases. The chatbot categorised these liver observations as LR-M, likely because it could not differentiate the nodular peripheral enhancement of

hemangiomas from the ring enhancement of LR-M. Sensitivity and specificity for LR-5 were, respectively, 0.8 and 0.96.

Appendix S3.

Performance of LiverAI against GPT-3.5 and GPT-4

The test dataset was submitted by one of the authors (J.P) into GPT-3.5 and GPT-4 through the web-browser interface (ChatGPT; OpenAI). Each report was introduced in a new chat session with the following prompt: "Which LI-RADS category would you assign to this radiologic report written in Spanish?" This evaluation was conducted in January 2024.

The results of this analysis are provided in Table S1. LiverAI outperformed GPT-3.5 and GPT-4 in all parameters related to LI-RADS categorisation and LR-5 identification (primary and secondary outcomes, respectively). For the simpler dichotomized malignancy approach, GPT-4 showed better accuracy and specificity for malignant observations compared to LiverAI. This discrepancy may be attributed to LiverAI's suboptimal performance in LR-3 categorisation, where 61.6% of the observations categorised as LR-3 by the ground truth were miscategorised as malignant by LiverAI. Despite this, we believe the results further validate LiverAI's performance, especially considering its high specificity for LR-5 identification (0.96) and perfect sensitivity for malignant observations (1.00).

Supplementary Figures

Figure S1. Schematic illustration of the development and function of the domain-specific chatbot LiverAI.

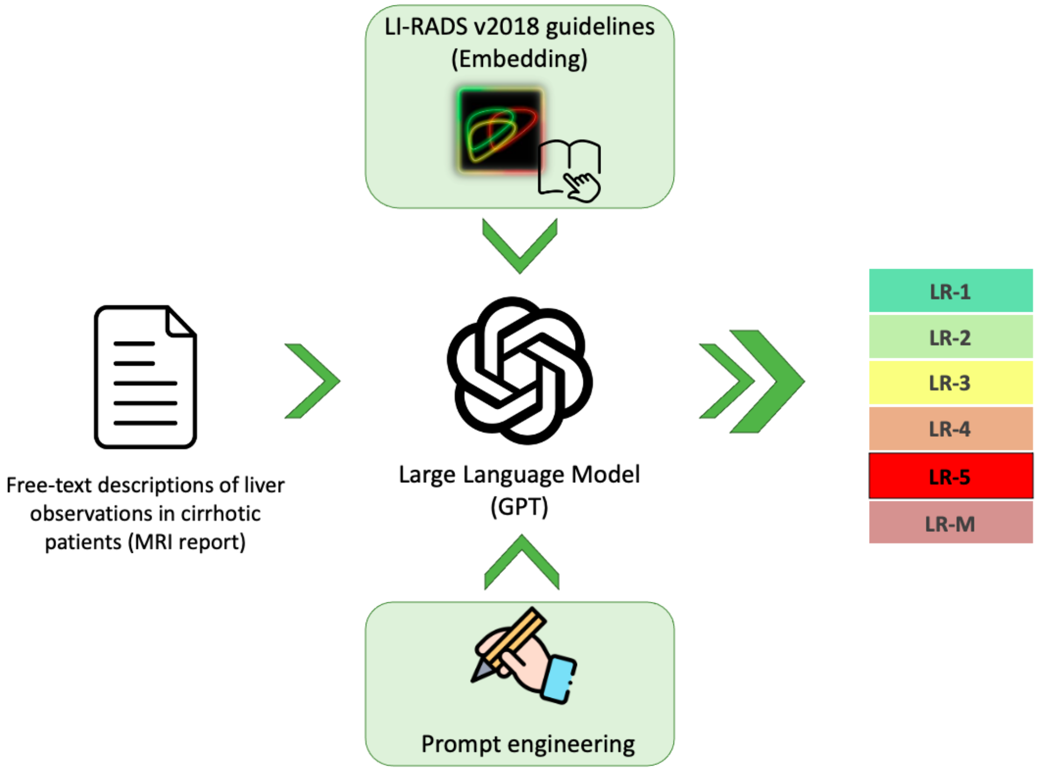

Supplementary Tables

Table S1. Performance statistics in the test dataset for GPT-3.5, GPT-4 and the domain-specific large language model (LiverAI).

LLM	LI-RADS		LR-5			Dichotomized LI-RADS		Malignancy	
	Accuracy	K value	Accuracy	Sensitivity	Specificity	Accuracy	K value	Sensitivity	Specificity
GPT-3.5	0.33 (0.25 – 0.43)	0.26 (0.16 – 0.37)	0.61 (0.52 – 0.69)	0.39 (0.29 – 0.51)	0.88 (0.79 – 0.96)	0.74 (0.65 – 0.81)	0.39 (0.21 – 0.56)	0.86 (0.77 – 0.94)	0.51 (0.35 – 0.66)
GPT-4	0.48 (0.41 – 0.58)	0.46 (0.37 – 0.56)	0.70 (0.61 – 0.77)	0.52 (0.40 – 0.62)	0.94 (0.87 – 1.00)	0.87 (0.81 – 0.93)	0.71 (0.58 – 0.85)	0.92 (0.86 – 0.98)	0.78 (0.67 – 0.90)
LiverAI	0.62 (0.55 – 0.71)	0.54 (0.42 – 0.65)	0.85 (0.80 – 0.91)	0.76 (0.69 – 0.86)	0.96 (0.91 – 1.00)	0.83 (0.75 – 0.89)	0.58 (0.42 – 0.73)	1.00 (1.00 – 1.00)	0.51 (0.37 – 0.66)

Data in parentheses are 95% confidence intervals.

Table S2. Examples of correct and incorrect categorisations made by the chatbot, translated from Spanish, alongside with the consensus agreement by the three radiologists.

Report	LiverAI	Consensus
A focal lesion measuring 13 mm in diameter is identified in segment VIII of the left hepatic lobe, which is hyperintense in T1 sequences and shows a loss of signal intensity in opposed-phase sequences. This lesion is isointense in T2 sequences and shows very discrete enhancement in the arterial phase of the dynamic study, being hypointense with a peritumoral pseudocapsule image in the portal and equilibrium phases.	LR-5	LR-5
A focal hepatic lesion is observed, which shows isosignal in sequences obtained under baseline conditions, which appears markedly hyperintense in the arterial phase of the dynamic study, and which returns to being isointense in relation to the adjacent hepatic parenchyma in the rest of the contrasted sequences. The enhancement observed in the arterial phase is relatively homogeneous. No peritumoral pseudocapsule is observed. The described lesion measures approximately 12 mm in size and is located in hepatic segment VI.	LR-4	LR-3
Liver of small size and markedly irregular contours in relation to chronic hepatopathy. The hepatogram is also heterogeneous in the different phases of the obtained study. A hypervascular focal hepatic lesion is observed, localized in segment VII, and appears faintly hypointense in images obtained with fat saturation in the equilibrium phase, where it presents a very faint peritumoral pseudocapsule. In this sequence, the lesion measures 14 mm in size.	LR-5	LR-5
In the lateral segment of the left hepatic lobe, a focal lesion measuring 16 mm in diameter is identified, hypointense in T1-weighted sequences and discretely hyperintense in T2-weighted sequences, which shows peripheral enhancement in the arterial phase of the dynamic study, appearing	LR-4	LR-M

hyperintense in the remaining portal and delayed phases.		
A nodular image measuring 1.1 cm in size is observed, located in segment VIII, isointense in T2-weighted sequences, which shows signal loss in sequences obtained with fat phase opposition. In the arterial phase, it shows a small punctate enhancement, appearing hypointense in portal and late phases.	LR-4	LR-5
A focal lesion of 11 mm located in segments V-VI, spontaneously hyperintense in T1, faintly hyperintense in T2, and showing diffusion restriction. In the dynamic study, it presents hyperenhancement in the arterial phase, without evident washout in late venous phases.	LR-M	LR-3
A 11 mm nodule located in segment V, between the right anterior and posterior portal branches, isointense in basal sequences, hypervascular in the arterial phase, and remains hyperintense in venous sequences.	LR-3	LR-3
The study shows the presence of several hyperintense nodular images in T1-weighted sequences and without fatty content, isointense in T2, without arterial enhancement, and stable compared to the previous MRI study. The largest lesion, located in the left hepatic lobe, measures 10 mm.	LR-3	LR-2
In the dynamic study, a hyperenhancing nodular image with a hypointense halo measuring 21 mm in size is observed, located in the anterior segment of the right lobe (segment 5). The image is not identified in subsequently obtained sequences.	LR-4	LR-4
Lesion measuring 1.5x2 cm located at the posterior edge of segment V, subcapsular, hyperintense in T2, with nodular peripheral early-phase enhancement that progresses over time.	LR-M	LR-1
